# Learning Spatio Temporal Tactile Features with a ConvLSTM for the Direction Of Slip Detection

**DOI:** 10.3390/s19030523

**Published:** 2019-01-27

**Authors:** Brayan S. Zapata-Impata, Pablo Gil, Fernando Torres

**Affiliations:** 1 Automatics, Robotics and Artificial Vision Research Group, Department of Physics, System Engineering and Signal Theory, University of Alicante, 03690 Alicante, Spain; pablo.gil@ua.es (P.G.); fernando.torres@ua.es (F.T.); 2 Computer Science Research Institute, University of Alicante, 03690 Alicante, Spain

**Keywords:** tactile processing, direction of slip, spatio-temporal feature learning, deep learning

## Abstract

Robotic manipulators have to constantly deal with the complex task of detecting whether a grasp is stable or, in contrast, whether the grasped object is slipping. Recognising the type of slippage—translational, rotational—and its direction is more challenging than detecting only stability, but is simultaneously of greater use as regards correcting the aforementioned grasping issues. In this work, we propose a learning methodology for detecting the direction of a slip (seven categories) using spatio-temporal tactile features learnt from one tactile sensor. Tactile readings are, therefore, pre-processed and fed to a ConvLSTM that learns to detect these directions with just 50 ms of data. We have extensively evaluated the performance of the system and have achieved relatively high results at the detection of the direction of slip on unseen objects with familiar properties (82.56% accuracy).

## 1. Introduction

Tactile sensing is a key sense for humans when carrying out manipulation tasks. It helps us to overcome problems such as finding objects in the dark, distinguishing materials or textures as well as keeping a stable grip when holding objects, among many other possibilities. The interest in the application of tactile sensors is consequently very desirable for humanoids [1] and its use is growing in the field of robotic manipulation [2]. These sensors can provide a wide range of information like surface features, material properties and acting forces during the contact experienced by the sensor, which are potentially useful for dealing with manipulation tasks [3].

One area in which tactile sensors are applied is object recognition [4]. For example, in Ref. [5], a robotic hand with tactile sensors in the fingers and the palm was trained to recognise 20 different objects using only the tactile information. More recently, a set of novel tactile descriptors for object recognition were proposed [6]. These descriptors were learnt using machine learning and showed their potential to be transferred in order to recognise new objects. Furthermore, the classification of materials is also being researched. In Ref. [7], the authors worked on the recognition of six identical objects made of six different materials using tactile sensors as the only source of information. In other works, such as that of [8], deep learning strategies were used to recognise 15 objects from tactile images obtained from a flexible tactile sensor mounted in an adaptive gripper.

In this work, we focus on another problem in which tactile sensing is a cornerstone: slip detection. Whenever a robotic hand grasps an object, it is fundamental for the robot to recognise whether the object is firmly grasped or is slipping from the hand. However, recognising this grasp state is generally not sufficient in robotic manipulation tasks. It is of greater value to obtain more information such as recognising the type of slippage and its direction, that is, whether the slip is translational (north, east, south, west) or rotational (clockwise, anti-clockwise). If the robot were able to obtain these details regarding the slip event, it could react accordingly to correct it, by either re-adjusting its fingers or moving the wrist or arm, among other strategies. Therefore, in this paper we work on the detection of these slip directions with tactile sensors. This is achieved using spatio-temporal tactile features and deep learning methods in order to learn them.

Our main contribution is an extensive evaluation of the usefulness of spatio-temporal tactile features when employed to detect the direction of slip. This evaluation is made by applying a new type of recurrent neural network (ConvLSTM [9]), which is highly specialised in modelling spatio-temporal data. We chose to learn both spatial and temporal tactile features for this task because it seems reasonable to believe that the direction of slip has characteristics that belong to both components: slips occur in time (temporal aspect) and take place on a particular area of the hand (spatial aspect).

## 2. Related Work

Literature has approached the task of slip detection with tactile sensing from different levels of complexity. The objective of most of the previous works has been to detect two basic grasp states: stable and slipping. Some approaches have focused on the detection of these states by analysing the signals emitted by the tactile sensor. In Ref. [10], the authors presented an approach in which the Break-Away Friction Ratio was used in order to predict slip. Although the method predicted slips in 4.2 ms, it required a previous exploration of the surface (5–7 s) in order to model its friction coefficients and it was only tested on three scenarios. Later, in Ref. [11], the authors proposed a three-stage method that amplified an input force signal, which was then rectified and passed through a threshold, in order to return a binary slip signal (slip/stable). Similarly, in Ref. [12], three different methods for detecting these states were compared. The first exploited friction cones, the second processed the tactile sensor with a bandwidth filter and the third consisted of a random decision tree that correlated tactile readings with grasp states. A similar work was carried out later in Ref. [13], which showed that machine learning algorithms, and more specifically neural networks, could not only learn to detect these two grasp states but could also distinguish between two types of slips (translational/rotational), without hand-picked features for processing the tactile data as happens with classic methods. Although these works showed interesting approaches for slip detection, the slip types that they were able to recognise had a low degree of complexity. Identifying complex slip types is of greater value for a robotic controller since it allows the system to perform re-grasping strategies based on that knowledge, as demonstrated in Ref. [14].

In the recent work described in Ref. [15], six slip types were learnt using a neural network. The authors specifically worked on the task of learning to classify slippage in four directions (west, east, north, south) and two touching states (touch, no touch). Nevertheless, the proposed methodology continued with the trend of processing tactile readings as signals using a classical neural network, in which tactile values were concatenated in arrays, thus losing the spatial information encoded in the distribution of the sensor taxels. In contrast, some authors have been working on a different approach: rather than interpreting tactile readings as classical signals, they process them as if they were images. For example, in Ref. [16], a Convolutional Neural Network (CNN) was trained to distinguish slippery grasps from stable ones. The authors used matrix-like tactile sensors (sensors in which taxels are arranged in a matrix-like distribution) and their readings were fed to the CNN as if they were images. This method has been also applied to non-matrix tactile sensors (sensors in which measuring points are not arranged as in a matrix) and also showed promising results for slip detection [17].

CNNs have proved to have an outstanding performance as regards solving computer vision problems thanks to their ability to learn spatial features [18]. However, they do not encode temporal characteristics explicitly. An approach to overcome this was presented recently [19]. The authors used CNNs to extract spatial features from single tactile readings, which were arranged in a matrix-like structure. In parallel, sequences of these tactile images were also arranged in 3D images, in which the third channel corresponded to different tactile images in time, and then fed to the CNN in an attempt to learn temporal features. Despite this, the system was only tested for the object recognition task, which does not appear to require an explicit temporal aspect. Moreover, there are machine learning techniques that are more suitable than a CNN as regards learning temporal features.

One example of a machine learning technique that learns temporal features is a Long Short-Term Memory Network (LSTM) [20]. Some authors have combined these networks with CNNs in order to learn temporal and spatial features [21]. In the cited work, a tactile sensor with an internal camera, in combination with an external camera pointing to the gripper, was used to detect slips. The authors proposed to calculate spatial features from the sensor camera and the external camera using a CNN that had already been trained in a computer vision task, and then concatenated the features to create a mixed feature vector that was passed in sequences to a LSTM. The LSTM was, therefore, in charge of learning temporal features from the stream of spatial features. Although, in this case, the system learnt temporal features, it required the combination of tactile sensing with a vision system, something that is not always possible. Moreover, the learning of spatial and temporal features was not coupled to this task.

One of the firsts works on the use of spatio-temporal tactile features for slip detection was presented in Ref. [22]. The authors proposed a descriptor that encoded sequences of tactile readings by extracting features from the raw tactile data and then pooling them over time, repeating the process at various scales. As a result, the system returned a descriptor that simultaneously encoded spatial and temporal features. In experimentation, this approach proved to be robust for both spatial and temporal variations. Later, [23] presented one of the first applications for slip detection using Convolutional LSTM (ConvLSTM) [9], a special case of LSTM with internal convolutions which were designed to learn spatio-temporal features. As in Ref. [21], a tactile sensor with an internal camera was used in order to capture tactile readings. The recorded images were fed to the ConvLSTM in sequences, thus enabling it to learn spatio-temporal features. These two approaches showed great performance on the slip detection task. However, they could only differentiate between two states: slippery or stable grasp.

Given the promising performance of spatio-temporal tactile features in the aforementioned works, we propose their use to learn the task of detecting the direction of slip. Our work is close to that of [23]. We also use a ConvLSTM but we analyse its performance in a more complex task: detecting the direction of slip, which requires learning to recognise more types of slips and not only slip/stable grips. In addition, we utilise a pure tactile sensor with just 24 sensing points rather than a camera-based sensor with a 30 × 30 image resolution. In these terms, our work is also close to that of [15] because we aim to solve a similar problem using the same tactile sensor. Nevertheless, we overcome their limitations by covering more slip states, including rotational slippage (both clockwise and anti-clockwise). Moreover, we carry out a more in-depth study of the input data, in addition to use a different learning structure, that is more suitable as regards learning spatio-temporal features.

## 3. Methodology

### 3.1. Tactile Sensor and Data Processing

**Figure 1:**
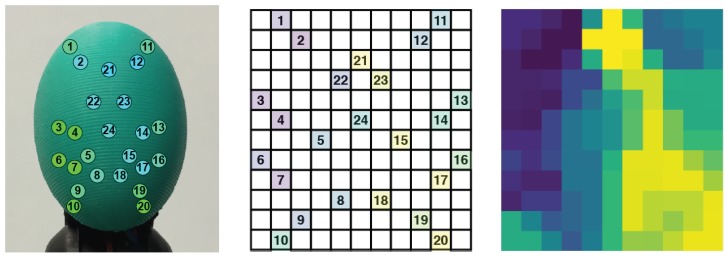
(**left**) Tactile sensor used in this work: the BioTac SP, which counts with 24 sensing points. (**middle**) Distribution of the BioTac SP electrodes in a 12 × 11 tactile image, proposed in [17]. (**right**) Result of filling the gaps in the tactile image with the mean value of the 8-closest neighbours. Images reproduced with permission from [17].

The tactile sensor used in this work is the BioTac SP [24], manufactured by Syntouch. This sensor is different from its previous version in that it has more electrodes and the circuits are integrated into just one single phalanx. The BioTac SP tactile sensor has a biomimetic design that provides three different sensory modalities, including force, pressure and temperature. In this work, we make use of only the force data. In more detail, the sensor holds 24 electrodes, distributed over the entire surface of its internal core, which record signals from 4 emitters and measure the impedance in the fluid that is between them and the elastic skin of the sensor. The fluid moves while contact is experienced by the sensor, thus affecting the measurements made by the electrodes. It additionally has a hydro-acoustic pressure sensor and a thermistor in order to detect vibrations and heat flows, among others. Figure 1 (left) shows a representation of the location of the electrodes in the sensor.

In order to learn features from the BioTac SP sensor, it is possible to build an array $\theta \in N^{1}$ that holds the readings from the 24 electrodes, such as $\theta =\{e_{1},e_{2},\ldots ,e_{24}\}$, where $e_{i}$ is the *i*-th electrode in Figure 1. If this array is read in consecutive *T* time steps, it is possible to create a sample $\Theta =\{\theta^{1},\theta^{2},\ldots ,\theta^{T}\}$ that holds *T* tactile readings. A learning algorithm would then be able to learn temporal features if sufficient Θ samples were collected, correctly labelling each Θ with one of the slip directions considered.

This 1D array θ is of less use as regards learning spatial features. It seems reasonable to believe that it is of greater value to use a different data structure that better encodes the spatial distribution of the electrodes in the sensor. A tactile image $\phi \in N^{2}$ can be created for this purpose. This is a 2D array in which the 24 electrodes values $e_{i}$ are spatially distributed to occupy the image pixels at certain coordinates (i,j). In a previous work [17], we studied how the 24 electrodes in the BioTac SP can be distributed in a tactile image in order to keep their local connectivity. In the present work, we use the best matrix distribution found in the cited work since it showed promising results in a similar task (grasp stability prediction). Basically, the tactile image ϕ consists of a 12 × 11 matrix in which the 24 electrodes are distributed as shown in Figure 1 (middle). All of the gaps—cells without assigned values—are then filled using the mean value of the 8-closest neighbours. By collecting multiple tactile images ϕ in consecutive *T* time steps, it is possible to create a sample $\Phi =\{\phi^{1},\phi^{2},\ldots ,\phi^{T}\}$ that could be used to learn spatio-temporal features.

### 3.2. LSTM Network

Long Short-Term Memory (LSTM) networks [20] are a special type of Recurrent Neural Networks (RNN). These neural networks, RNNs, have units whose output is connected not only to the next layer but also to the unit itself as an input. The neural network, therefore, behaves in a temporal manner that is appropriate as regards learning sequential models or time series [25,26].

LSTMs expand this models by including a new element in the neurons called the memory cell $c_{t}$. The purpose of this cell is to act as an accumulator of previous inputs $x_{t-1}$, thus enabling the unit to remember the information it has already processed. Through the use of gates, the unit can decide whether it should accumulate the new input $x_{t}$ in the memory cell $c_{t}$, and also whether it should forget the previous state $c_{t-1}$ and should propagate the memory cell $c_{t}$ to the output $h_{t}$ (hidden state). We use this network in this work as a baseline model for learning to detect the direction of slip. The key equations of the implementation used are shown in Equation (1):

(1) $$\begin{array}{c} \begin{array}{cc} i_{t} & =\sigma (W_{i}x_{t}+b_{ii}+U_{i}h_{t-1}+b_{hi})\\ f_{t} & =\sigma (W_{f}x_{t}+b_{if}+U_{f}h_{t-1}+b_{hf})\\ g_{t} & =tanh(W_{g}x_{t}+b_{ig}+U_{g}h_{t-1}+b_{hg})\\ o_{t} & =\sigma (W_{o}x_{t}+b_{io}+U_{o}h_{t-1}+b_{ho})\\ c_{t} & =f_{t}\circ c_{t-1}+i_{t}\circ g_{t}\\ h_{t} & =o_{t}\circ tanh\left(c_{t}\right) \end{array} \end{array}$$

where σ is the sigmoid function, tanh is the hyperbolic tangent function, $i_{t}$ is the input gate, $f_{t}$ is the forget gate, $g_{t}$ is the cell gate, $o_{t}$ is the output gate, $h_{t-1}$ is the hidden state at the previous time step t−1 or the initial hidden state at time 0, $c_{t-1}$ is the previous cell state at time t−1, $h_{t}$ is the hidden state at time *t*, $c_{t}$ is the cell state at time *t*, $x_{t}$ is the input at time *t*, *W* and *U* are the learnable weights and *b* are the bias terms. Here, ∘ denotes the Hadamard or entry-wise product.

**Figure 2:**
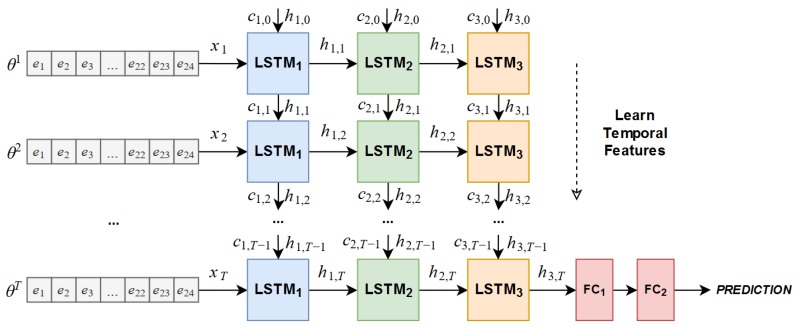
Architecture of the Long Short-Term Memory (LSTM) network used as baseline for comparison.

This type of neural network has been applied in previous works in order to tackle sequence modelling problems like natural language processing [27] and scene labelling [28], among others. In this work, we use the LSTM as a baseline model for comparison since it is an algorithm that is used to learn temporal features. The architecture tested during experimentation is shown in Figure 2.

### 3.3. ConvLSTM Network

Convolutional LSTM (ConvLSTM) [9] is an extension of LSTM networks designed to handle spatio-temporal data. In a ConvLSTM, instead of using 1D vectors, all of the inputs $x_{t}$, cell outputs $c_{t}$, hidden states $h_{t}$ and gates $i_{t},f_{t},g_{t},o_{g}$ are 3D tensors whose last two dimensions are rows and columns. By doing this, the ConvLSTM learns spatial features from pictures while simultaneously learning temporal ones. Some changes were introduced to the LSTM equations in order to create the ConvLSTM:

(2) $$\begin{array}{c} \begin{array}{cc} i_{t} & =\sigma (W_{i}*x_{t}+U_{i}*h_{t-1}+V_{i}\circ c_{t-1}+b_{i})\\ f_{t} & =\sigma (W_{f}*x_{t}+U_{f}h_{t-1}+V_{f}\circ c_{t-1}+b_{f})\\ g_{t} & =tanh(W_{g}*x_{t}+U_{g}h_{t-1}+b_{g})\\ o_{t} & =\sigma (W_{o}*x_{t}+U_{o}h_{t-1}+V_{o}\circ c_{t-1}+b_{o})\\ c_{t} & =f_{t}\circ c_{t-1}+i_{t}\circ g_{t}\\ h_{t} & =o_{t}\circ tanh\left(c_{t}\right) \end{array} \end{array}$$

where “∗” denotes a convolutional operator and *V* is a new set of learnable weights. The rest of the notation is kept from Equation (1). The changes made, therefore, focus on both the dimensionality of the parameters learnt and the input. Furthermore, the ConvLSTM takes into account previous cell state $c_{t-1}$ to calculate each gate value.

**Figure 3:**
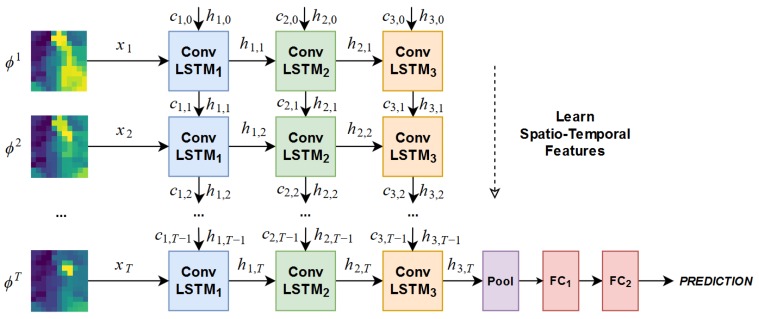
Architecture of the ConvLSTM network tested in experimentation.

These networks are relatively new so there are few previous works on their application. The original authors [9] used it to forecast prediction using radar images. These networks have recently been tested on slip detection [23] but only to distinguish a stable from slippery grasp, as commented on in Section 2. We propose to use ConvLSTM networks to learn a more complex task, as well as to detect the direction of slip. As a result, we have experimented with the architecture shown in Figure 3.

### 3.4. Data Acquisition

**Figure 4:**
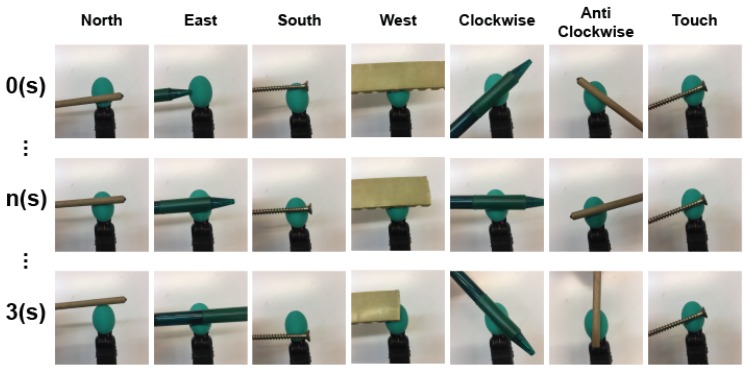
Examples of the data acquisition methodology: We manually performed the movement over the sensor for each of the considered classes.

When creating Θ samples (temporal-only data) or Φ samples (spatio-temporal data) from the BioTac SP, we need to record a dataset of tactile readings and label them. In this work, our goal is learn to detect 7 directions of slip: slip north, slip west, slip south, slip east, rotate clockwise, rotate anti-clockwise and stable contact (there is contact without movement). In order to generate the dataset, we performed each of the movements on one BioTac SP sensor using different objects. The procedure followed to record a sequence of data for each category is shown in Figure 4. We maintained different velocities and produced different forces in order to increase the variability of the recorded sequences. Each recorded sequence held a movement of only one of the categories, which was acquired by saving the values of the electrodes for 3 s. Since the sensor publishes its data with a peak frequency of 100 Hz, we had approximately 300 consecutive tactile readings for each of the 24 electrodes from each sequence.

**Figure 5:**
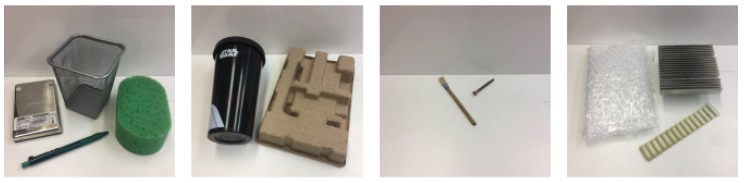
We recorded 4 datasets (**left** to **right**): Basic set, Solids set, Small objects set and Textures set.

Following this methodology, we recorded 4 datasets containing different objects and textures (see Figure 5). The Basic object set comprises 4 objects: a metal pen holder, a hard drive, a sponge and a plastic pen. The Solids set comprises 2 objects: a plastic bottle and a piece of cardboard. The Small set consists of 2 objects: A wooden brush and a metal screw. Finally, the Textures set comprises 3 objects: a striped piece of plastic, a piece of bubble wrap and a striped heat sink. These objects were selected by taking into account their dimensions, textures and materials, in order for them to produce different pressure patterns in the BioTac SP sensor. We decided to split them into 4 sets in order to later check the generalisation capabilities of the proposed method. For each direction of slip, we recorded 4 sequences for every object in order to keep the datasets balanced. Table 1 provides further information about the datasets, which we leave freely available at https://github.com/yayaneath/BioTacSP-DoS.

**Figure 6:**
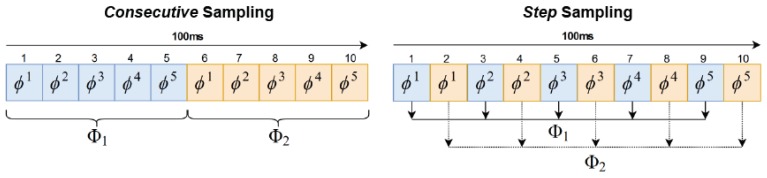
Example of the sampling methods used in this work in order to create training samples: (**left**) *Consecutive* sampling and (**right**) *Step* sampling.

During experimentation, we tested two means of sampling these tactile readings in order to create training samples. On the one hand, we built input samples in which the tactile images were consecutive recorded readings. This is, a sample Φ held *T* tactile images that followed each other in time, like $\Phi =\{\phi^{t},\phi^{t+1},\phi^{t+2},\ldots ,\phi^{t+(T-1)}\}$. We called this method *Consecutive* sampling. On the other hand, we also tested a method in which the tactile readings were selected with steps *s*. Hence, a sample Φ that held *T* tactile images with a reading step *s* resulted in $\Phi =\{\phi^{t},\phi^{t+1s},\phi^{t+2s},\ldots ,\phi^{t+(T-1)s}\}$. This method was called *Step* sampling. Note that *Consecutive* sampling is a special case of *Step* sampling with s=1. Figure 6 shows a simplified example of both sampling methods on a sequence of 100 ms (10 tactile readings). Both methods have T=5 in the example and the *Step* sampling uses s=2.

Finally, since the range of values returned by the sensor is in the range [0,3488], where lower values mean higher pressure, we normalised them before training the models. They were rescaled to the range [0,1] using Equation (3). In the case of θ samples, this was applied to the 24 values $e_{i}$. However, in the case of ϕ samples, the equation was applied to every pixel in the tactile image.

(3) $$x^{\prime }=\frac{x-min(X)}{max(X)-min(X)}$$

where $x^{\prime }$ is the rescaled value, *x* is the previous value and min(X) and max(X) are the minimum and maximum values in the data distribution *X*.

## 4. Experimentation

During experimentation, we first analysed the performance of the ConvLSTM learning spatio-temporal features from the recorded tactile data. Afterwards, we compared the generalisation capabilities of this algorithm with the LSTM network by training with the Basic set and testing on each of the other three sets. All of the experiments in this section were run on Ubuntu 16.04, Python 3.5.2, CUDA 8.0 and PyTorch 0.4.1 on a computer equipped with an Intel i5-4690K CPU at 3.5 GHz (4 cores), 16 GiB DDR3 RAM at 1867 MHz and a GeForce GTX 960 GPU.

### 4.1. ConvLSTM Parameters

In this first set of experiments, we exhaustively studied how the performance of the ConvLSTM changes depending on a number of parameters: number of ConvLSTM layers, the size of the convolutional filters and the number of filters inside each ConvLSTM layer. All of these experiments were carried out following a 5-fold cross validation methodology with the Basic set. The performance metric used to report results in this section is the accuracy. All of the trained networks used the cross-entropy loss and the Adam optimizer with a learning rate equal to 0.001.

**Figure 7:**
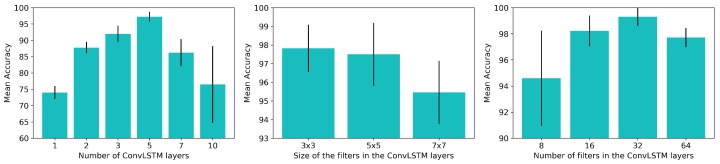
Achieved performance varying: (**left**) number of ConvLSTM layers, (**middle**) size of the filters and (**right**) number of filters in the layers.

We defined an architecture similar to that previously shown in Figure 3. The number of ConvLSTM layers was tuned on the first test, after which we changed the size of the convolutional filters and eventually tuned the number of filters in the ConvLSTM layers in order to choose that which provided the best performance. However, the rest of the network was fixed during the experiments: the last ConvLSTM layer was connected to a pooling layer, which was connected to two fully-connected layers with 32 units and ReLU activations. We also fixed the length of the sequence of the training samples to 10 consecutive tactile readings, the batch size to 32 and the number of epochs to 30. Figure 7 shows the results obtained from these experiments concerning the tuning of these parameters.

As can be seen in Figure 7 (left), as the number of ConvLSTM layers in the network increased from 1 to 5, the system performed better in the same amount of training iterations. Nevertheless, increasing the number of ConvLSTM layers any further was detrimental to the performance. The system achieved an accuracy of 97.23% with 5 layers, but this rate decreased to 76.48% when the network had 10 ConvLSTM layers. As more layers were added, the network became more complex and it was more difficult to converge in the same amount of epochs. Given the results, we chose to keep using 5 ConvLSTM layers for the following experiments since this configuration had the best accuracy rate, with a low standard deviation (97.23%±1.46%).

We performed three experiments with three different sizes for the convolutional filters in the ConvLSTM layers (Figure 7 (middle)): 3 × 3, 5 × 5 and 7 × 7. The input samples Φ had a size equal to [B,T,C,H,W], where *B* was the batch size (32), *T* was the length of the sequence (10), *C* was the number of channels (1, since we were using one sensor), *H* was the height of the tactile image (12) and *W* was the width of the tactile image (11). As can be seen in Figure 7 (middle), the performance that was achieved decreased as we increased the size of the filter. Since the input tactile images were 12 × 11 images, bigger filters covered wider areas of the image. The filters were not, therefore, focusing on patches of the image but were rather taking into account almost the whole tactile image. Consequently, in the following experiments we continued to use 3 × 3 filters, thus allowing the network to pay more attention to lower-level details in the tactile images.

Finally, we tested the networks with 8, 16, 32 and 64 filters per ConvLSTM layer (Figure 7 (right)). As had occurred with the first test in which we changed the number of ConvLSTM layers, as we increased the number of filters in these layers, the complexity of the network increased and it was, therefore, more difficult for it to learn the problem in the same number of epochs. In addition, increasing the number of ConvLSTM layers and the number of filters in them also increased the number of parameters in the network, signifying that these more complex networks were more time and memory consuming. As a result, in the following experiments, we chose to use ConvLSTM layers with 32 filters because they had the best accuracy rates in our experiments (99.30%±0.71%).

### 4.2. Temporal Input Sequence

**Figure 8:**
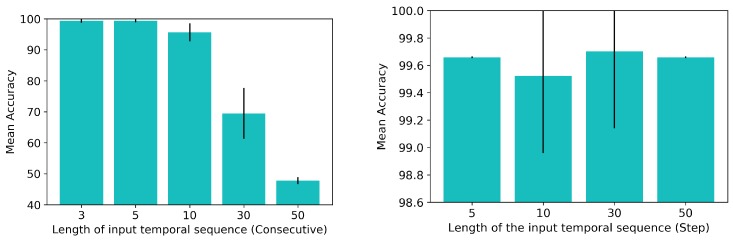
Achieved performance varying the input to the ConvLSTM network: (**left**) *Consecutive* tactile readings and (**right**) tactile readings with *Step* s=5.

For these experiments, we changed the length of the input samples in order to check how this would affect the performance of the ConvLSTM network. This was done by training network with 5 ConvLSTM layers (32 filters of 3 × 3 per layer), followed by a pooling layer and two fully-connected layers with 32 units and ReLU activations. Again, the experiments were carried out with a 5-fold cross-validation on the Basic set. The cross-entropy loss was used as well as the Adam optimizer with a learning rate equal to 0.001. The batch size was equal to 32 and 30 epochs were run. Figure 8 shows the performance achieved when varying the input sequence in these experiments.

Figure 8 (left) presents the results obtained from the experiments with the *Consecutive* sampling method, using T=[3,5,10,30,50]. Since the BioTac SP sensor has a peak publishing frequency of 100 Hz, these tests checked the performance of the ConvLSTM with approximately 30 ms, 50 ms, 100 ms, 300 ms and 500 ms of consecutive readings. Using 3 and 5 consecutive readings yielded accuracy rates of 99.31%±0.56% and 99.37%±0.49%, respectively. However, further increasing the number of tactile images in the samples reduced the accuracy rate achieved by the system.

Noise could be the reason for this decrease in the performance of the system with an increase in the consecutive tactile images included in each sample. In experimentation, we found that the BioTac SP sensor does not maintain the values of the readings at a constant number, but instead they fluctuate continuously (up to 197 points in its range [0,3488]). The ConvLSTM memory could be affected by this noise and might require more training epochs in order to learn it. With just 3 or 5 consecutive readings there is less fluctuation, so the network can accurately learn to detect the direction of slip.

Regarding the *Step* sampling experiments, we tested T=[5,10,30,50] and s=5. Since the peak recording frequency was 100 Hz, this value of *s* meant an approximate time gap of 50 ms between a tactile reading $\phi^{i}$ and the next $\phi^{i+s}$. Figure 8 (right) presents the results obtained after running these experiments. In all of these tests, the performance of the system was above 99% accuracy, the standard deviation being the major difference between them. The ConvLSTM network managed to learn samples better when using the *Step* method than when using the *Consecutive* method. Since tactile readings ϕ were more distant in time from each other, noise was not a big issue for the network and the direction of slip was more clearly identified in these samples.

Although these results suggested that using the *Step* sampling method would be better, we had to take into account the drawback of this method: time. A *Step* sampling with T=5 and s=5 meant that in order to create a sample Φ we had to wait 250 ms until enough tactile readings ϕ were published by the BioTac SP sensor. The accuracy rate achieved by this configuration was 99.66%±0.006%. The best result achieved by the tests made with the *Consecutive* method was obtained using T=5, which yielded 99.37%±0.49%. However, creating a sample Φ with the *Consecutive* method only required 25 ms: the *Consecutive* method gave us a similar result in terms of accuracy in a tenth part of the time required to create a sample with the *Step* method. We found that this was of paramount importance for the problem in hand. Due to the fact that we want to detect the direction of slip, the earlier we can detect it, the faster we can react and avoid a complete loss of grip on the object. We consequently continued to use the *Consecutive* sampling method with T=5 in the following experiments.

### 4.3. Convergence

**Figure 9:**
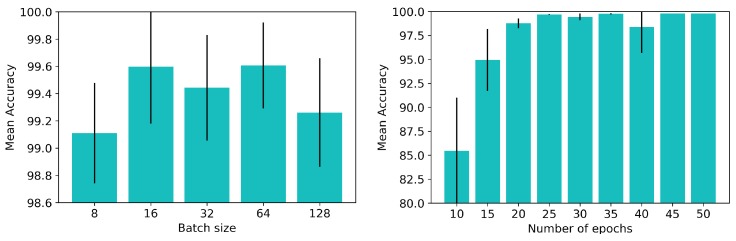
Achieved performance varying (**left**) the batch size and (**right**) the number of epochs.

We checked how batch size and the number of epochs would affect the convergence of the ConvLSTM network. In detail, we used the same network as in the previous section, trained with input samples whose length was 5 consecutive tactile readings. Again, the experiments were carried out with a 5-fold cross-validation on the Basic set. The cross-entropy loss was used as well as the Adam optimizer with a learning rate equal to 0.001. We present the obtained results in Figure 9.

Regarding the batch size, there were no significant differences in the accuracy rate depending on this parameter. All of the configurations tested achieved rates above 99% with similar standard deviations. Literature states [29] that using larger batches results in models with lower generalisation capabilities. We, therefore, chose to use a batch size of 32 training samples for the rest of the experiments in order to keep a good balance between the training speed and the quality of the resulting model. Finally, the tests carried out with the number of epochs showed that at around 30 epochs the ConvLSTM network converged, and that training further did not lead to an improvement in the accuracy rate. The remaining experiments carried out were consequently run until 30 epochs.

### 4.4. Robustness Test

In these experiments, we verified how the ConvLSTM, trained with the Basic set, managed to generalise in order to detect the direction of slip in samples recorded from the other three sets: the Solids set, the Small set and the Textures set. In addition, we compared its performance with a tuned LSTM network, since it computes temporal features while the ConvLSTM computes spatio-temporal features, which should be more useful for the problem tackled in this work.

**Figure 10:**
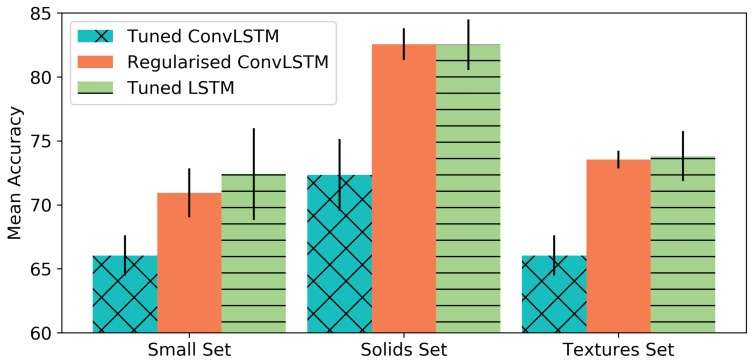
Mean accuracy obtained by the Tuned ConvLSTM, the Regularised ConvLSTM and the Tuned LSTM trained on the Basic set and tested with the Small, Solids, and Textures sets.

Two ConvLSTM networks were used to carry out these experiments: the *Tuned ConvLSTM*, which is the best performing network from the previous experiments with the Basic set; and the *Regularised ConvLSTM*, which is a simpler network with higher generalisation capabilities. In detail, the *Tuned ConvLSTM* consists of a network with 5 ConvLSTM layers (32 filters of 3 × 3) followed by a pooling layer and two fully-connected layers with 32 units and ReLU activations. With regard to *Regularised ConvLSTM*, it has 3 ConvLSTM layers (32 filters of 3 × 3) followed directly by two fully-connected layers with 32 units and ReLU activations, so there is no pooling after the ConvLSTM layers. In addition, this network is regularised with batch normalisation, dropout in the fully-connected layers (30% probability of a unit to be zeroed) and L2 regularisation (0.001). Finally, the *Tuned LSTM* has a similar configuration to the *Regularised ConvLSTM*, but instead of the ConvLSTM layers it has 3 LSTM layers with 32 units. All of these networks were trained with a batch size of 32 samples during 30 epochs, using cross-entropy loss and the Adam optimizer with a learning rate equal to 0.001. The results were obtained after training the networks 5 times, shuffling the training set (Basic set) and testing them each time with the Solids, Small and Textures set. Figure 10 presents the accuracy rates achieved by each of the networks for each test set.

Figure 10 shows that the network that performed worst in this robustness test was the Tuned ConvLSTM. This network was configured by choosing the parameters that performed best in previous sections. However, those tests were performed on the Basic set, which did not guarantee that the resulting network could properly generalise to new objects and textures. As a result, the Tuned ConvLSTM overfitted to the Basic set. In contrast, the Regularised ConvLSTM was prepared in order to avoid overfitting with regularisation techniques, in addition to reducing the network complexity. As a result, this ConvLSTM performed much better than the tuned version.

The Regularised ConvLSTM achieved a greater mean accuracy on the Solids set than on the other two sets: it yielded an accuracy rate of 82.56% for this set, while it achieved accuracy rates of 70.94% and 73.54% for the Small and Textures sets, respectively. This trend was also visible in the results obtained for the Tuned ConvLSMT and the Tuned LSTM. The Solids and the Basic sets are similar in that both sets contain objects with similar features: the bottle and the cardboard in the Solids set have a similar stiffness and size to the hard drive and the metal pen holder in the Basic set. As a result, the Solids set produced contact patterns that were similar to the ones already seen by the networks in the Basic set. In contrast, the Small set and the Textures set contain objects that are far more unlike the objects in the Basic set. The features learnt from the Basic set with these networks were consequently not as good at classifying samples from these two sets as they were at classifying samples from the Solids set. Table 2 shows further details of the performance of the networks trained on the test sets.

Since the problem we approach in this work is a multi-class classification, the arithmetic mean of the precision, recall and F1-score among all of the classes is reported, along with the mean accuracy, in Table 2. With regard to the performance of the Tuned LSTM, it yielded similar results to the Regularised ConvLSTM. Generally speaking, the Tuned LSTM obtained greater values in most of the sets and metrics than the Regularised ConvLSTM, particularly for the Small set tests. Nevertheless, the differences were not significant. Moreover, the standard deviations of its results were also greater for all the metrics, showing that the Tuned LSTM had a less robust performance. The Regularised ConvLSTM had a more constant performance during the 5 iterations of training and testing carried out. It was less sensitive to the training so its spatio-temporal features were stable. That is, the training iterations of the Regularised ConvLSTM resulted in similar spatio-temporal features that performed similarly. The temporal features calculated for the different training iterations of the Tuned LSTM were more dissimilar to each other, and it was for this reason that its performance varied more from training iteration to training iteration. The most significant difference between the LSTM and the two ConvLSTM approximations was the input data type. In contrast to the LSTM, which received the values of the 24 electrodes, the ConvLSTM received a created tactile image whose size might have been small for this type of neural network. In deep learning, in order to learn better spatial features, images with higher resolution than our tactile images are frequently used for training. Despite this fact, the Regularised ConvLSTM achieved a robust performance on the generalisation tests.

**Figure 11:**
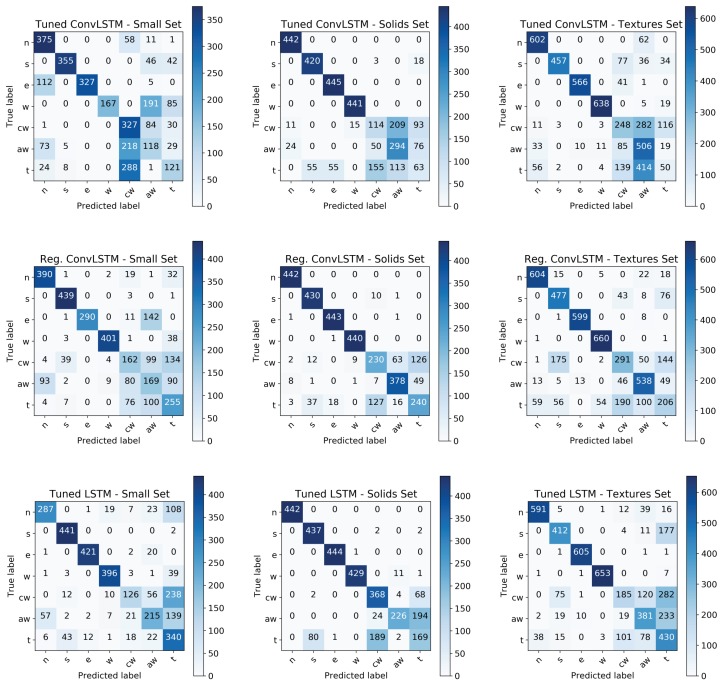
Confusion matrix of the best performing network from the 5 training iterations: (**top row**) Tuned ConvLSTM, (**middle row**) Regularised ConvLSTM and (**bottom row**) Tuned LSTM. *n* stands for slip north, *s* for slip south, *e* for slip east, *w* for slip west, cw for clockwise rotational slip, aw for anti-clockwise rotational slip and *t* for touch (stable contact).

Figure 11 shows the confusion matrices of the best performing configurations of each network for each test set. Note that distinguishing the four translational directions of slip was not as hard as distinguishing clockwise rotational slip, anti-clockwise rotational slip and touch (labelled *cw*, *aw*, *t* in the matrices). Samples belonging to the four translational slips were, in general, correctly classified but samples belonging to the rotational slips and touch were frequently confused. More specifically, these three classes were confused with each other. In the rotational slips, there was no translational movement over the entire surface of the sensor. Hence, the tactile readings were localised in an area of it but there was some fluctuation in values around the centre of the rotation. Touch samples were produced by placing the object at a certain point on the surface of the sensor without moving it. Given that the readings from the BioTac SP sensor are constantly wavering owing to its inherent noise, it could be possible that a touch sample had a similar fluctuating pattern as regards the values of the electrodes to that produced by a rotational slip. This would be confirmed by the fact that rotational slips were mainly confused with touch and not significantly with each other.

## 5. Conclusions

The task of detecting the direction of slip using tactile sensors is still an open issue in robotic dexterous manipulation. Previous works approach this problem by simplifying it to the classification of grasps in two states: stable or slippery. However, it is of greater use if a robotic controller gets to know not only whether or not a grip is stable, but also the direction towards which a grasped object could be slipping. In this work we, therefore, propose the use of spatio-temporal features learnt using a specialised neural network: a Convolutional Long Short-Term Memory (ConvLSTM).

In order to check the performance of this type of networks in the task of detecting the direction of slip, we used a BioTac SP sensor. This sensor has 24 sensing points or taxels, which are electrodes that are distributed over the entire surface of the sensor. Since the ConvLSTM uses a matrix-like structure to learn spatio-temporal features, we converted the tactile readings from the BioTac SP sensor into tactile images. Four object sets, containing a total of 11 different objects, were used to capture a new tactile dataset, available at https://github.com/yayaneath/BioTacSP-DoS. Samples recorded were classified in slip north, slip south, slip east, slip west, slip clockwise, slip anti-clockwise or touch.

After training in experimentation with these datasets, we have proved that ConvLSTM can learn useful spatio-temporal features in order to detect the direction of slip effectively. In the task of detecting these seven states on already seen objects, the system achieved an accuracy rate of 99%. To do so, the system required only an input with five consecutive tactile readings, which were recorded in just 50 ms of wall time. In addition, a forward-pass of a batch took only 3 ms. As a result, the proposed approach proved to be fast and accurate for this task.

However, the ConvLSTM network was sensitive to new objects. During the robustness experiments, its performance dropped to an accuracy rate of 82.56% in the case of new objects with familiar properties (Solids set), but continued to decrease to 73.54% and 70.94% for stranger sets like the Textures and Small sets. In comparison, a tuned LSTM attained similar results with these tests, though with a greater sensitivity to the training convergence. In addition, the problem in hand proved to be challenging and the networks struggled to distinguish rotational slips from stable contacts (touch).

In the future, we would like to implement the proposed system in the control loop of a multi-fingered hand, thus enabling it to use this information to manage re-grasping strategies. Moreover, we would like to integrate more tactile sensors in the detection of the direction of slip. It seems reasonable to believe that doing so should increase the generalisation capabilities of the system since more sensing points would reduce the entropy of slips, like the rotational ones recorded.

## Figures and Tables

**Table 1:** Size of the recorded datasets used in experimentation.

Object Set	Items	Sequences/Class	Total Sequences	Total Tactile Readings
Basic	4	4	112	30,673
Solids	2	4	56	15,492
Small	2	4	56	15,520
Textures	3	4	84	22,662

**Table 2:** Mean and standard deviation of the accuracy, precision, recall and F1-score for each of the tested models on each of the test sets.

Object Set	Small Set			Solids Set			Textures Set		
Network	Tuned ConvLSTM	Regularised ConvLSTM	Tuned LSTM	Tuned ConvLSTM	Regularised ConvLSTM	Tuned LSTM	Tuned ConvLSTM	Regularised ConvLSTM	Tuned LSTM
**Mean** **Accuracy (%)**	57.44±2.41	70.94±1.92	72.41±3.58	72.34±2.80	82.56±1.24	82.51±1.97	66.04±1.57	73.54±0.68	73.80±1.95
**Mean** **Precision (%)**	66.36±4.43	72.35±4.01	75.49±4.13	68.26±5.56	80.93±3.29	82.58±3.67	68.01±6.39	72.03±2.82	74.73±4.18
**Mean** **Recall (%)**	57.42±7.11	70.73±6.60	72.79±9.06	72.31±3.89	82.27±2.83	82.69±6.71	66.49±7.41	73.72±2.10	74.13±7.85
**Mean** **F1-Score (%)**	58.50±5.00	70.90±4.86	72.67±5.48	68.80±4.29	81.08±2.48	81.84±4.29	66.37±5.52	72.46±2.11	73.51±4.13
